# Interim [^18^F]FDG PET/CT can predict response to anti-PD-1 treatment in metastatic melanoma

**DOI:** 10.1007/s00259-020-05137-7

**Published:** 2020-12-18

**Authors:** Christos Sachpekidis, Annette Kopp-Schneider, Leyun Pan, Dimitrios Papamichail, Uwe Haberkorn, Jessica C. Hassel, Antonia Dimitrakopoulou-Strauss

**Affiliations:** 1 Clinical Cooperation Unit Nuclear Medicine, German Cancer Research Center (DKFZ), Im Neuenheimer Feld 280, 69210 Heidelberg, Germany; 2 Department of Biostatistics, German Cancer Research Center (DKFZ), Heidelberg, Germany; 3 Department of Nuclear Medicine, University of Heidelberg, Heidelberg, Germany; 4 Department of Dermatology and National Center for Tumor Diseases (NCT), University Hospital Heidelberg, Heidelberg, Germany

**Keywords:** Metastatic melanoma, Anti-PD-1 therapy, Immunotherapy, Treatment response evaluation, Interim [^18^F]FDG PET/CT, PERCIMT criteria, EORTC criteria

## Abstract

**Purpose:**

In an attempt to identify biomarkers that can reliably predict long-term outcomes to immunotherapy in metastatic melanoma, we investigated the prognostic role of [^18^F]FDG PET/CT, performed at baseline and early during the course of anti-PD-1 treatment.

**Methods:**

Twenty-five patients with stage IV melanoma, scheduled for treatment with PD-1 inhibitors, were enrolled in the study (pembrolizumab, *n* = 8 patients; nivolumab, *n* = 4 patients; nivolumab/ipilimumab, 13 patients). [^18^F]FDG PET/CT was performed before the start of treatment (baseline PET/CT) and after the initial two cycles of PD-1 blockade administration (interim PET/CT). Seventeen patients underwent also a third PET/CT scan after administration of four cycles of treatment. Evaluation of patients’ response by means of PET/CT was performed after application of the European Organization for Research and Treatment of Cancer (EORTC) 1999 criteria and the PET Response Evaluation Criteria for IMmunoTherapy (PERCIMT). Response to treatment was classified into 4 categories: complete metabolic response (CMR), partial metabolic response (PMR), stable metabolic disease (SMD), and progressive metabolic disease (PMD). Patients were further grouped into two groups: those demonstrating metabolic benefit (MB), including patients with SMD, PMR, and CMR, and those demonstrating no MB (no-MB), including patients with PMD. Moreover, patterns of [^18^F]FDG uptake suggestive of radiologic immune-related adverse events (irAEs) were documented. Progression-free survival (PFS) was measured from the date of interim PET/CT until disease progression or death from any cause.

**Results:**

Median follow-up from interim PET/CT was 24.2 months (19.3–41.7 months). According to the EORTC criteria, 14 patients showed MB (1 CMR, 6 PMR, and 7 SMD), while 11 patients showed no-MB (PMD). Respectively, the application of the PERCIMT criteria revealed that 19 patients had MB (1 CMR, 6 PMR, and 12 SMD), and 6 of them had no-MB (PMD). With regard to PFS, no significant difference was observed between patients with MB and no-MB on interim PET/CT according to the EORTC criteria (*p* = 0.088). In contrary, according to the PERCIMT criteria, patients demonstrating MB had a significantly longer PFS than those showing no-MB (*p* = 0.045). The emergence of radiologic irAEs (*n* = 11 patients) was not associated with a significant survival benefit. Regarding the sub-cohort undergoing also a third PET/CT, 14/17 patients (82%) showed concordant responses and 3/17 (18%) had a mismatch of response assessment between interim and late PET/CT.

**Conclusion:**

PET/CT-based response of metastatic melanoma to PD-1 blockade after application of the recently proposed PERCIMT criteria is significantly correlated with PFS. This highlights the potential ability of [^18^F]FDG PET/CT for early stratification of response to anti-PD-1 agents, a finding with possible significant clinical and financial implications. Further studies including larger numbers of patients are necessary to validate these results.

**Supplementary Information:**

The online version contains supplementary material available at 10.1007/s00259-020-05137-7.

## Introduction

Metastatic melanoma is a highly aggressive tumor, largely refractory to existing therapies, and associated with a very poor prognosis [1]. While until lately the treatment options for metastatic melanoma were limited, the recent development and introduction in clinical practice of several novel immunotherapeutic agents as well as of targeted therapy with BRAF and MEK inhibitors have revolutionized the systemic treatment of the disease, leading to unprecedented response and survival rates of melanoma patients [2].

The main form of immunotherapy applied in this new era of melanoma management involves immune checkpoint blockade. This immunomodulatory approach activates the immune system against tumors through the binding of the cytotoxic T lymphocyte–associated protein 4 (CTLA-4) and/or the programmed cell death protein 1 (PD-1), both of which are expressed by T cells [3, 4]. The monoclonal antibody ipilimumab, which acts by blocking CTLA-4, is considered a landmark agent in this context, being the first immunotherapeutic drug demonstrating a clear benefit in survival of patients with advanced melanoma, which led to its approval by the US Food and Drug Administration (FDA) and the European Medicines Agency (EMA) in 2011 [5]. A few years later, a second class of immune checkpoint inhibitors (ICIs), the PD-1 inhibitors nivolumab and pembrolizumab, were also approved for the treatment of melanoma, after having shown survival benefit in these patients [6–8]. Moreover, the anti-PD-1 monoclonal antibodies have shown superiority over ipilimumab, leading to their application both as single agents and in combination with ipilimumab, which is nowadays seldom used as monotherapy [9–13].

Despite these dramatic improvements, a significant amount of patients—approximately 40–45%—show no response to immunotherapy [14]. Additionally, the mechanism of action of these agents (which is markedly different from usual cytotoxic approaches—notably by generating inflammations rather than direct lysis) can pose relevant challenges in the interpretation of treatment response by conventional imaging approaches [15]. Furthermore, several patients experience a “new class” of cumulative, dose-dependent, and sometimes life-threatening side effects, the immune-related adverse events (irAEs), which scope is wide [16]; importantly, the occurrence of such irAEs may be of prognostic value, revealing a response to immunotherapy [17, 18]. These issues raise the question of how to evaluate the response to ICIs in a reliable fashion and early in the course of treatment. This information would help discriminate responders from non-responders, offering significant therapeutic and prognostic implications in the entire spectrum of patient management. Unfortunately, there exist at present only few reliable predictors of long-term response to immunotherapy.

[^18^F]FDG PET/CT is considered the elective imaging technique in detecting metastatic disease in advanced melanoma [19–23]. Moreover, a growing amount of recently published literature has highlighted the potential role of the modality in the prediction of treatment response to ICIs in melanoma, rendering it an attractive tool for the monitoring of immunotherapy [24–31].

In quest of identifying reliable biomarkers for the prediction of long-term outcomes to immunotherapy, we aim in the present prospective study to assess the value of interim [^18^F]FDG PET/CT performed after the first two cycles of anti-PD-1 treatment.

## Materials and methods

### Patients

Twenty-five patients (12 males, 13 females; mean age 54.7 years) with unresectable, stage IV melanoma undergoing immunotherapy with PD-1 inhibitors applied either as monotherapy (pembrolizumab, nivolumab) or as combination treatment with CTLA-4 inhibitors (nivolumab/ipilimumab) were enrolled in the study (Table 1). Pembrolizumab was administered intravenously at a dose of 2 mg/kg every 3 weeks, and nivolumab was administered intravenously at a dose of 3 mg/kg every 2 weeks. The combination ICI therapy was administered as an induction of 4 cycles of nivolumab (1 mg/kg) and ipilimumab (3 mg/kg) every 3 weeks, followed by single-agent nivolumab administration (3 mg/kg) every 2 weeks. The included patients had not received chemotherapy for at least 1 month prior to the initial PET/CT studies. None of the patients had a history of diabetes. Patients gave written informed consent to participate in the study and to have their medical records released. The study was approved by the Ethical Committee of the University of Heidelberg and the Federal Agency for Radiation Protection (Bundesamt für Strahlenschutz).

**Table 1:** Patient characteristics

Patient number	Age	Gender	LDH at baseline (U/I)	Previous treatment	ICI treatment	EORTC PET response	PERCIMT PET response	Radiologic signs of irAEs	Progression	PFS (months)
1	56	F	770	Chemosaturation with melphalan, pembrolizumab, gemcitabine/treosulfan	Nivolumab/ipilimumab	PMD (no-MB)	PMD (no-MB)	Thyroiditis	Yes	1.5
2	34	F	247	Pembrolizumab, IMCgp100	Nivolumab/ipilimumab	CMR (MB)	CMR (MB)	Bone marrow activation, colitis	No	17.9
3	46	F	166	Vemurafenib/cobimetinib	Nivolumab/ipilimumab	PMD (no-MB)	SMD (MB)	Duodenitis	Yes	4.0
4	54	M	218	Nivolumab	Nivolumab/ipilimumab	PMR (MB)	PMR (MB)	Colitis	Yes	3.2
5	53	M	275	No	Nivolumab/ipilimumab	SMD (MB)	SMD (MB)	-	Yes	0.4
6	50	F	344	Vemurafenib/cobimetinib	Nivolumab/ipilimumab	SMD (MB)	SMD (MB)	Bone marrow activation, lymphadenopathy	No	19.3
7	59	M	204	No	Nivolumab/ipilimumab	PMR (MB)	PMR (MB)	Sarcoid-like reaction, arthitis	No	38.7
8	44	F	340	Dabrafenib/trametinib	Nivolumab/ipilimumab	PMR (MB)	PMR (MB)	Bone marrow activation, colitis	No	18.3
9	60	F	269	No	Nivolumab/ipilimumab	PMR (MB)	PMR (MB)	–	No	21.2
10	48	F	246	No	Nivolumab/ipilimumab	PMD (no-MB)	SMD (MB)	Lymphadenopathy	No	24.2
11	55	F	224	No	Nivolumab	SMD (MB)	SMD (MB)	-	Yes	6.2
12	84	F	195	No	Pembrolizumab	SMD (MB)	SMD (MB)	-	Yes	11.0
13	79	M	205	No	Pembrolizumab	PMD (no-MB)	PMD (no-MB)	Arthritis	Yes	2.0
14	20	F	186	No	Nivolumab/ipilimumab	PMD (no-MB)	PMD (no-MB)	-	Yes	17.8
15	52	M	275	No	Pembrolizumab	PMR (MB)	PMR (MB)	-	No	42.1
16	52	M	170	No	Pembrolizumab	PMD (no-MB)	SMD (MB)	-	Yes	14.6
17	53	F	260	No	Nivolumab	SMD (MB)	SMD (MB)	-	Yes	9.6
18	65	M	201	Ipilimumab	Pembrolizumab	SMD (MB)	SMD (MB)	-	Yes	1.9
19	67	F	290	No	Pembrolizumab	PMD (no-MB)	SMD (MB)	Bone marrow activation, colitis	Yes	5.9
20	55	M	200	Ipilimumab/nivolumab, dabrafenib/trametinib	Pembrolizumab	PMD (no-MB)	PMD (no-MB)	-	Yes	1.5
21	58	M	183	Pembrolizumab	Nivolumab/ipilimumab	PMR (MB)	PMR (MB)	Gastritis, colitis	Yes	17.0
22	50	M	195	No	Nivolumab/ipilimumab	PMD (no-MB)	PMD (no-MB)	-	Yes	12.1
23	80	M	364	No	Pembrolizumab	PMD (no-MB)	PMD (no-MB)	-	Yes	0.9
24	47	F	256	Dabrafenib	Nivolumab	SMD (MB)	SMD (MB)	-	Yes	1.1
25	47	M	271	Ipilimumab, dabrafenib	Nivolumab	PMD (no-MB)	SMD (MB)	-	Yes	2.7

*F*, female; *M*, male; *LDH*, lactate dehydrogenase; *EORTC*, European Organization for Research and Treatment of Cancer; *PERCIMT*, PET Response Evaluation Criteria for IMmunoTherapy; *CMR*, complete metabolic response; *PMR*, partial metabolic response; *SMD*, stable metabolic disease; *PMD*, progressive metabolic disease; *irAEs*, immune-related adverse events; *PFS*, progression-free survival

### [^18^F]FDG PET/CT data acquisition

[^18^F]FDG PET/CT was performed before the start of treatment (baseline PET/CT) and after the initial two cycles of ICIs’ administration (interim PET/CT) in all 25 patients. Moreover, 17 patients of the cohort also had a third PET/CT scan within 2 weeks after administration of four cycles of treatment.

Patients underwent a whole body PET/CT after intravenous administration of maximum 250 MBq [^18^F]FDG 60 min post-injection (p.i.). Imaging was performed from the head to the feet with an image duration of 2 min per bed position. A dedicated PET/CT system (Biograph mCT, S128, Siemens Co., Erlangen, Germany) with an axial field of view of 21.6 cm with TruePoint and TrueV, operated in a three-dimensional mode was used. A low-dose attenuation CT (120 kV, 30 mA) was used for attenuation correction of the PET data and for image fusion. All PET images were attenuation-corrected and an image matrix of 400 × 400 pixels was used for iterative image reconstruction. Iterative image reconstruction was based on the ordered subset expectation maximization (OSEM) algorithm with two iterations and 21 subsets as well as time of flight (TOF).

### [^18^F]FDG PET/CT data analysis

Data analysis consisted of visual (qualitative) assessment of the PET/CT scans and semi-quantitative evaluation based on standardized uptake value (SUV) calculations. PET/CT images were analyzed on an Aycan workstation by three nuclear medicine physicians (CS, DP, ADS). Images were interpreted by consensus. Visual analysis was based on the identification of sites of focal, non-physiologic [^18^F]FDG uptake above surrounding background activity, which were considered consistent with melanoma lesions.

Moreover, patterns of [^18^F]FDG uptake on interim PET/CT suggestive of radiologic manifestations of irAEs to immunotherapy were documented. Based on our experience and the published literature in the field [29, 32], we defined radiologic irAEs as sites of newly emerging, increased compared to baseline imaging, non-malignant [^18^F]FDG accumulation in organs known to exhibit immune-related signs on PET/CT. In particular, a new, diffusely enhanced tracer uptake in organs such as the gastrointestinal tract (mostly colon), the thyroid gland and the bone marrow, or, respectively, a new, relatively symmetrical, increased uptake in lymph nodes (e.g., mediastinal/hilar, inguinal) and in joints following ICIs were considered suggestive of radiologic irAEs in these organs. Semi-quantitative evaluation was based on volumes of interest (VOIs) and on subsequent calculation of SUV_mean_ and SUV_max_. VOIs were drawn using the pseudo-snake algorithm of the Pmod software (http://www.pmod.com/files/download/v31/doc/pbas/4729.htm) and were placed over melanoma lesions.

### Response evaluation

Evaluation of patients’ response by means of [^18^F]FDG PET/CT was performed after application of the European Organization for Research and Treatment of Cancer (EORTC) 1999 criteria [33] as well as the recently proposed PET Response Evaluation Criteria for IMmunoTherapy (PERCIMT) [26]. Both criteria classify tumor response into 4 categories. The major difference between these criteria lies in the number of newly emerging lesions between baseline and follow-up PET/CT for the characterization of PMD: according to EORTC, the appearance of one new hypermetabolic lesion leads to patient classification to PMD, but according to PERCIMT, this requires the appearance of a minimum of four new lesions below 1 cm or respectively ≥ 3 new lesions of 1.0–1.5 cm or ≥ 2 new lesions of more than 1.5 cm. Another difference between the criteria is the role of SUV, which is central in EORTC, while it is not taken into account in PERCIMT (Table 2).

**Table 2:** Summary of the EORTC and PERCIMT response criteria

	EORTC	PERCIMT
CMR	Complete resolution of [^18^F]FDG uptake within the tumor volume	Complete resolution of all pre-existing [^18^F]FDG avid lesions. No new, [^18^F]FDG avid lesions.
PMR	Decrease in tumor SUV > 25% after more than 1 therapeutic cycle	Complete resolution of some pre-existing [^18^F]FDG avid lesions. No new, [^18^F]FDG avid lesions.
SMD	Increase in tumor SUV < 25% or decrease in SUV < 15%	Neither PMD nor PMR/CMR
PMD	Increase in tumor SUV > 25% or appearance of new lesions	≥ 4 new lesions of less than 1 cm in functional diameter or ≥ 3 new lesions between 1.0–1.5 cm in functional diameter or ≥ 2 new lesions of more than 1.5 cm in functional diameter

*EORTC*, European Organization for Research and Treatment of Cancer; *PERCIMT*, PET Response Evaluation Criteria for IMmunoTherapy; *CMR*, complete metabolic response; *PMR*, partial metabolic response; *SMD*, stable metabolic disease; *PMD*, progressive metabolic disease; *SUV*, standardized uptake value

Stable disease (SD) represents a satisfactory outcome following immunotherapy, since—in contrast to conventional chemotherapy—it can be durable and survival rates related to SD are comparable to those associated with response [34–36]. Based on this, patients were further grouped into two groups: those demonstrating metabolic benefit (MB), including patients with SMD, PMR, and CMR, and those demonstrating no MB (no-MB), including patients with PMD.

### Statistical analysis

Progression-free survival (PFS) was measured from the date of interim PET/CT until disease progression or death from any cause. Kaplan-Meier estimates were generated and median PFS estimated. Median follow-up time was determined by inverse Kaplan-Meier estimation. For univariate comparison of PFS, a log-rank test was used. Statistical analysis was performed using R version 4.0.2 (The R Foundation for Statistical Computing 2020) and R packages survival and prodlim. The results were considered significant for *p* values less than 0.05 (*p* < 0.05).

## Results

### Patient cohort

All included patients received treatment with anti-PD-1 agents, applied either as monotherapy (pembrolizumab, *n* = 8 patients; nivolumab, *n* = 4 patients) or as combination therapy (nivolumab/ipilimumab, *n* = 13 patients). The mean baseline serum lactate dehydrogenase (LDH) was 262 U/l with four patients having pathologically high LDH levels and 21 of them normal levels. The detailed characteristics of the studied patients are presented in Table 1.

### PET/CT findings

**Fig. 1:**
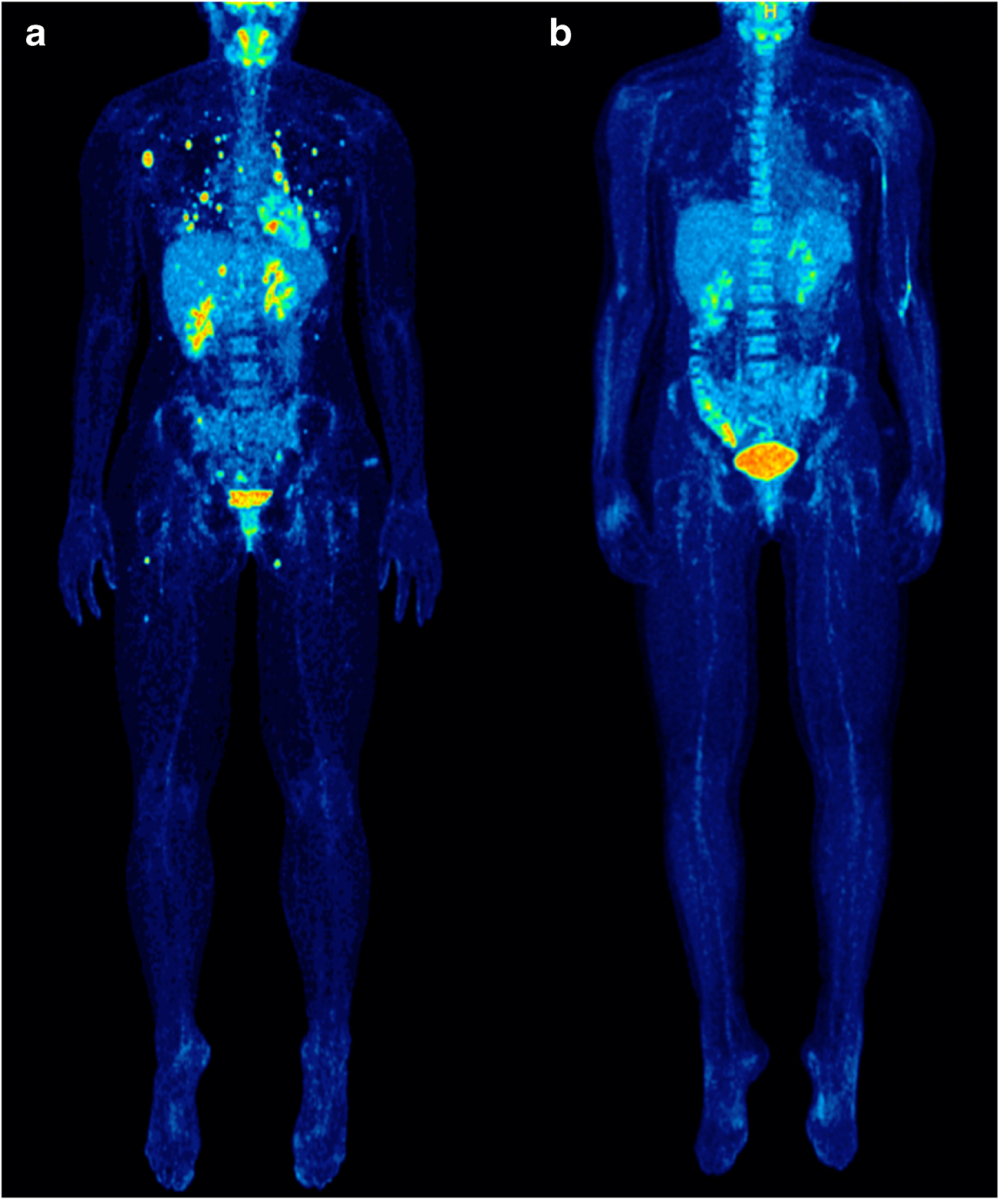
Maximum intensity projection (MIP) [^18^F]FDG PET/CT images of a 34-year-old woman with metastatic melanoma before initiation of immunotherapy with nivolumab/ipilimumab (A) and after administration of two cycles of treatment (B). Baseline PET/CT image shows multiple lymph node, pulmonary, hepatic, adrenal, soft tissue, and osseous metastases (A). Interim PET/CT shows a complete metabolic remission (CMR) of all baseline lesions. The patient demonstrated metabolic benefit (MB) according to both the EORTC and PERCIMT criteria (B). Moreover, diffusely increased [^18^F]FDG uptake is observed in the ascending colon and the bone marrow on interim PET/CT. At the time of writing, the patient was still progression-free having reached a PFS of 17.9 months

The findings of interim PET/CT were compared to those of the baseline scan and PET/CT-based response evaluation was performed for all 25 patients. According to the EORTC criteria, 14 patients showed MB (1 CMR, 6 PMR, and 7 SMD), while 11 patients showed no-MB (PMD). Respectively, the application of the PERCIMT criteria revealed that 19 patients had MB (1 CMR, 6 PMR, and 12 SMD), while six of them had no-MB (PMD) (Table 3) (Fig. 1).

**Table 3:** Summary of the patients’ classifications in different response groups based on interim PET/CT and according to the EORTC and PERCIMT response criteria (*n* = 25 patients)

	Metabolic benefit			No-metabolic benefit
	CMR	PMR	SMD	PMD
EORTC	1	6	7	11
PERCIMT	1	6	12	6

*EORTC*, European Organization for Research and Treatment of Cancer; *PERCIMT*, PET Response Evaluation Criteria for IMmunoTherapy; *CMR*, complete metabolic response; *PMR*, partial metabolic response; *SMD*, stable metabolic disease; *PMD*, progressive metabolic disease

**Fig. 2:**
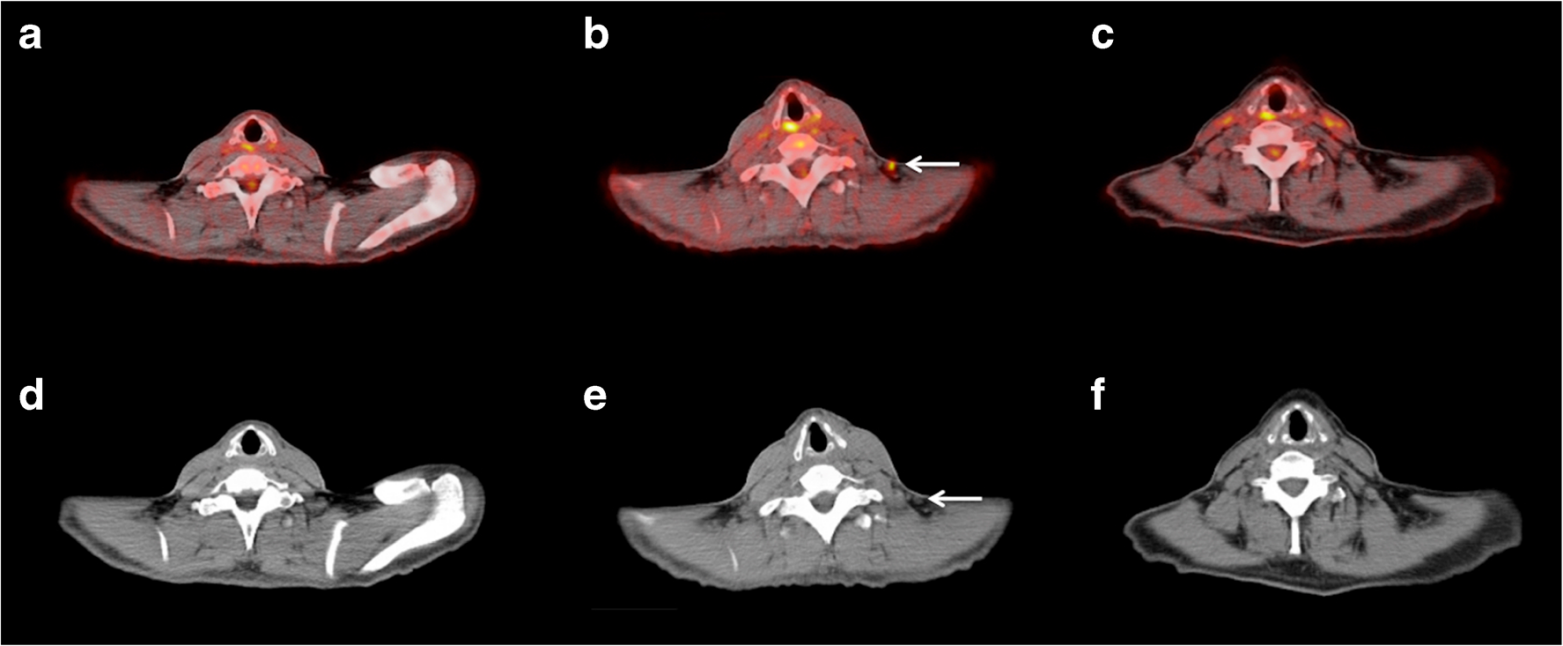
Transaxial PET/CT (upper row, A–C) and low-dose CT (lower row, D–F) images at the cervical level of a 48-year-old female patient with advanced melanoma. The PET/CT (A) and CT (D) images obtained before immunotherapy show no pathologic lesions. Interim PET/CT performed after two cycles of nivolumab/ipilimumab shows a new [^18^F]FDG-avid lymph node (white arrow; B, E), suspicious of metastatic involvement. According to the EORTC criteria, the patient showed progressive metabolic disease (PMD), while according to PERCIMT, he had stable metabolic disease (SMD). A third PET/CT obtained after administration of four cycles of nivolumab/ipilimumab shows remission of the lesion (C, F), suggesting pseudoprogression of the cervical finding on interim PET/CT. The patient had a PFS of 24.2 months and was still progression-free at last contact

With regard to the subgroup of 17 patients undergoing three PET/CT examinations (baseline, interim, late), the following results were revealed for interim PET/CT: 11 patients had MB (1 CMR, 5 PMR, and 5 SMD) and six patients had no-MB (PMD) according to EORTC, while 14 patients had MB (1 CMR, 5 PMR, and 8 SMD) and three of them had no-MB (PMD) according to PERCIMT. Respectively, on late PET/CT imaging, 13 patients had MB (3 CMR, 5 PMR, and 5 SMD) and four patients had no-MB (PMD) according to both EORTC and PERCIMT (Table 4). Two of the three patients exhibiting a mismatch between EORTC (PMD, no-MB) and PERCIMT (SMD, MB) on interim PET/CT, finally showed MB on the third examination based on both criteria (pseudoprogression) (Fig. 2). Respectively, one patient with early signs of PMD (no-MB) according to EORTC and SMD (MB) according to PERCIMT eventually exhibited PMD (no-MB) on the third examination based on both criteria.

**Table 4:** Summary of the patients’ classifications in different response groups based on interim (after two cycles of ICIs) and late (after four cycles of ICIs) PET/CT (*n* = 17 patients)

		EORTC				PERCIMT			
		MB (late PET/CT)			No-MB (late PET/CT)	MB (late PET/CT)			No-MB (late PET/CT)
		CMR	PMR	SMD	PMD	CMR	PMR	SMD	PMD
1.MB (interim PET/CT)	CMR	1	0	0	0	1	0	0	0
	PMR	0	5	0	0	0	5	0	0
	SMD	1	0	0	4	2	0	5	1
No-MB (interim PET/CT)	PMD	1	0	1	4	0	0	0	3

*EORTC*, European Organization for Research and Treatment of Cancer; *PERCIMT*, PET Response Evaluation Criteria for IMmunoTherapy; *MB*, metabolic benefit; *no-MB*, no metabolic benefit; *CMR*, complete metabolic response; *PMR*, partial metabolic response; *SMD*, stable metabolic disease; *PMD*, progressive metabolic disease

In total, 11 patients had PET/CT findings suggestive of irAEs on interim PET/CT, the majority of whom were under combination treatment: nine patients received nivolumab and ipilimumab, while two of them were under pembrolizumab (*p* = 0.015). In particular, the most common radiologic adverse event was a diffusely increased [^18^F]FDG uptake in the colon, defined as colitis, which was observed in five patients. One of these five patients also developed severe diarrhea, as clinical sign of treatment-induced colitis. Other gastrointestinal tract radiologic irAEs included gastritis (*n* = 1 patient) and duodenitis (*n* = 1 patient), defined as diffuse increased tracer uptake in the stomach and duodenum, respectively. Moreover, arthritis, defined as diffuse increased, periarticular, symmetrical tracer uptake in joints, was observed in two patients, and thyroiditis, a diffuse increased uptake in the thyroid gland, was observed in one patient. Furthermore, reactive, increased, symmetrical uptake in lymph nodes was observed in three patients, one of whom exhibited a sarcoid-like lymphadenopathy. Finally, diffuse increased bone marrow uptake was seen in four patients representing bone marrow activation in terms of a systemic immune response [37] (Table 1).

### Survival analysis

**Fig. 3:**
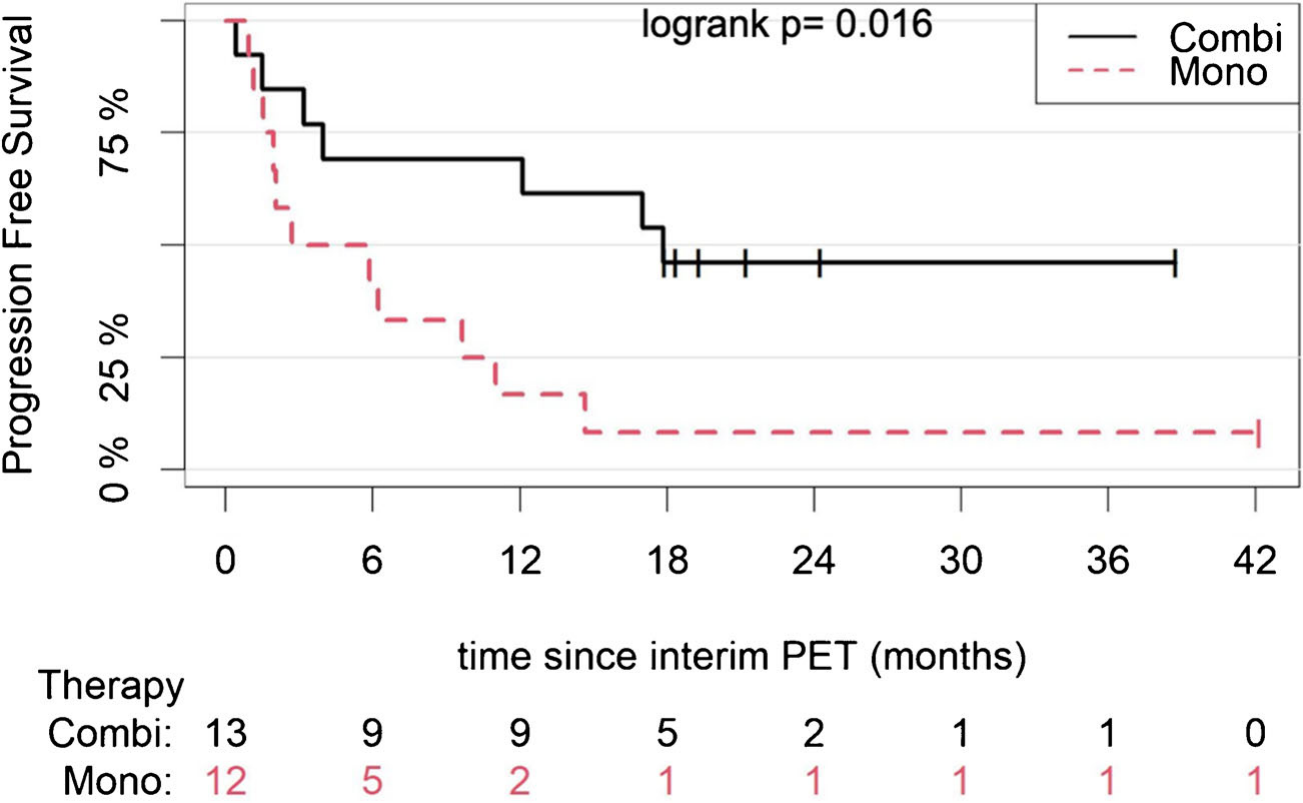
Kaplan-Meier estimates of PFS according to the anti-PD-1 treatment applied. The numbers of patients at risk in each group and for the respective time points are shown below the plots. Combi, combination therapy (ipilimumab/nivolumab); Mono, monotherapy (nivolumab or pembrolizumab)

Median follow-up (95% CI) of the patient cohort from interim PET/CT was 24.2 months (19.3–41.7 months). Patients receiving combination treatment (nivolumab/ipilimumab) had a median PFS of 17.8 months (4.0–NA), while those receiving PD-1 blockade monotherapy (nivolumab or pembrolizumab) had a median PFS of 4.3 months (1.9–NA) (*p* = 0.016) (Fig. 3).

**Fig. 4:**
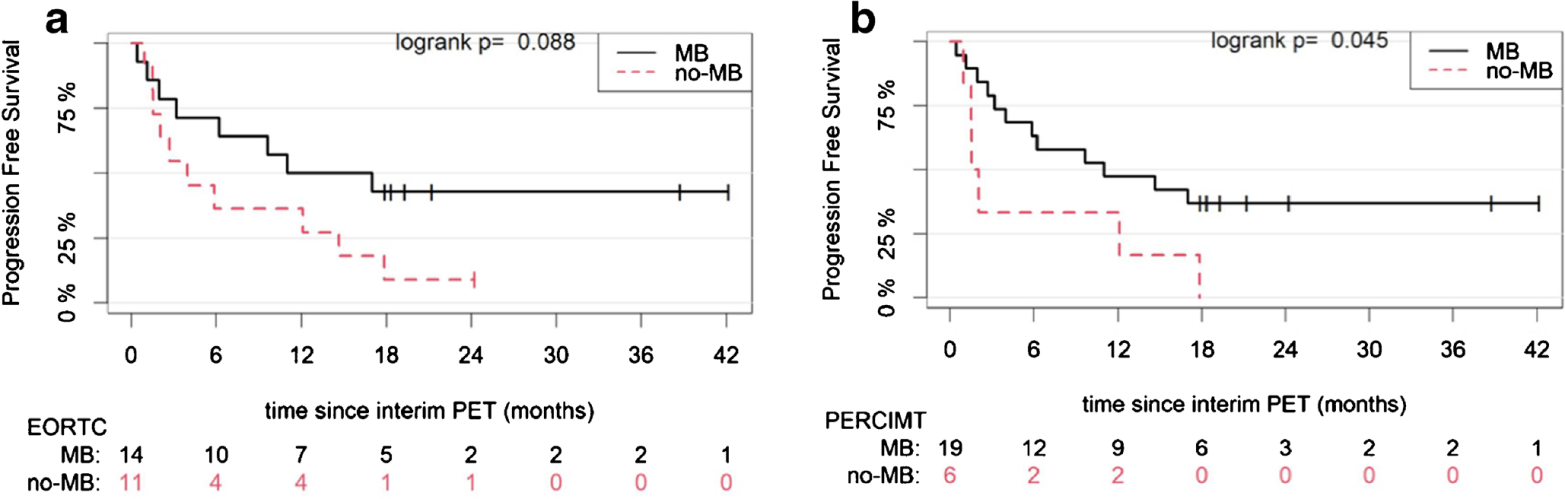
Kaplan-Meier estimates of PFS according to the EORTC (A) and the PERCIMT (B) criteria. The numbers of patients at risk in each group and for the respective time points are shown below the plots. MB, metabolic benefit; no-MB, no metabolic benefit

Based on the EORTC criteria, patients with MB on interim PET/CT had a median PFS of 14.0 months (6.2–NA), while those with no-MB had a median PFS of 4.0 months (2.0–NA) (*p* = 0.088) (Fig. 4A). Respectively, according to the PERCIMT criteria, patients with MB had a median PFS of 11.0 months (5.9–NA), while those with no-MB had a median PFS of 1.8 months (1.5–NA) (*p* = 0.045) (Fig. 4B).

**Fig. 5:**
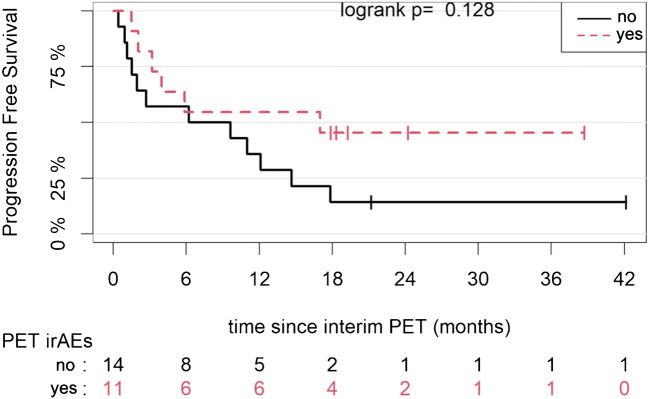
Kaplan-Meier estimates of PFS according to the emergence of radiologic irAEs on interim PET/CT. The numbers of patients at risk in each group and for the respective time points are shown below the plots

The patient cohort was further dichotomized on the basis of the emergence of radiologic irAEs on interim PET/CT. Patients with irAEs had a median PFS of 17.0 months (4.0–NA), and patients without irAEs had a median PFS of 7.9 months (1.9–NA) (*p* = 0.128) (Fig. 5).

Finally, the potential correlation of baseline LDH with PFS was also investigated. However, pathologic levels of LDH had no adverse effect on survival of the cohort (*p* = 0.642) (Supplementary File 1).

## Discussion

The therapeutic benefit of immune checkpoint blockade of PD-1 and CTLA-4 in the treatment of metastatic melanoma is variable [38]. Our understanding of how ICIs affect T cell evolution is incomplete [39], limiting the ability to derive full clinical benefit and, moreover, to predict responses from these drugs. However, tracking early response to immunotherapy is key for treatment options.

In this study, we investigated the role of interim PET/CT, performed after application of two cycles of anti-PD-1 treatment, in prediction of survival of metastatic melanoma patients. Our results showed that tumor response as classified by the PERCIMT criteria is significantly correlated with PFS, with metabolic responders demonstrating a significant survival benefit over non-responders. Respectively, the application of the EORTC criteria also led to a higher PFS for metabolic responders compared to non-responders but this difference was not statistically significant. These findings highlight the ability of [^18^F]FDG PET/CT—in particular, after application of the recently introduced PERCIMT criteria—to monitor and predict response to anti-PD-1 agents at an early but clinically relevant time point. This is of particular importance in clinical decision-making and prediction of outcome early during the course of immunotherapy, rendering PET/CT a potentially significant tool for the management of these patients. The main strengths of our study include its prospective nature, its rigorous protocol with imaging performed at strictly defined time points during treatment, the standardized for all patients PET/CT procedure, and the correlation with survival analysis.

Hitherto, a non-negligible number of studies have evaluated the efficacy of PET/CT in predicting treatment response of metastatic melanoma patients to ICIs. While most papers have focused on later time points during the course or after the end of treatment [26, 28, 30–32, 40], few of them also reported on application of the imaging modality early during immunotherapy. Our group previously showed in a cohort of 22 patients that PET/CT performed after two ipilimumab cycles—and after application of the EORTC criteria—correctly predicted treatment response after completion of the 4-cycle treatment in the majority (87%) of PMD patients and in all SMD patients [24]. In an expanded analysis of the ipilimumab patient cohort (*n* = 41 patients), the capacity of interim PET/CT in predicting clinical benefit to the agent was also highlighted. In that analysis, the performance of the PERCIMT criteria was superior to that of EORTC, which is in line with the results of the present study [27]. Furthermore, Cho et al. studied 20 melanoma patients treated with different ICIs (anti-PD-1 and anti-CTLA-4) with PET/CT at 3–4 weeks into therapy and found that a combination of changes in lesional dimensions along with changes in [^18^F]FDG uptake is a more accurate predictor of eventual response than each of these parameters alone. The authors proposed the PET/CT criteria for early prediction of response to immune checkpoint inhibitor therapy (PECRIT) criteria, based on a combination of the response evaluation criteria in solid tumors (RECIST) and PET response criteria in solid tumors (PERCIST) [25].

The current analysis represents the first study focusing on the role of interim PET/CT, performed as early as after application of two cycles of PD-1 blockade, in survival prediction of metastatic melanoma patients undergoing treatment with this class of ICIs. Taken together, the herein presented findings as well as those of previous studies in the field build further evidence on the potentially significant role of PET/CT performed early during the course of immunotherapy for prediction and stratification of response to treatment.

Novel patterns of response and progression, not previously seen with conventional therapies (such as cytotoxic or targeted anticancer regiments), have been described under immunotherapy and are attributed to the unique mechanism of action of these agents. In particular, phenomena such as pseudoprogression and irAEs may render response assessment to ICI challenging, questioning the utility of imaging modalities.

Evaluation of response to immunotherapy by means of PET/CT is primarily visual and subjective in nature. Considering the aforementioned challenges raised by the advent of immunotherapy for imaging interpretation, our group has recently introduced the PERCIMT criteria in metastatic melanoma, in an attempt to meet the need for reliable response assessment based on PET/CT. The cornerstone of these criteria is the finding that the absolute number of newly emerged [^18^F]FDG-avid lesions is more predictive of clinical outcome than SUV changes [26]. In specific, the application of a threshold of four newly emerged lesions on post-therapy PET/CT scan—with a decreasing cutoff of lesion number as the functional diameter of the lesions increases—in a cohort of 41 patients could predict clinical benefit to treatment with the agent ipilimumab better than the standard threshold of one new lesion or an increase in SUV, conventionally applied with the EORTC criteria. This was also confirmed in the present analysis with a significant correlation of metabolic response on interim PET with PFS only after application of PERCIMT.

Pseudoprogression, defined as an initial increase of tumor burden before the disease responds to therapy, has been initially described in melanoma patients undergoing ipilimumab therapy [41]. Since this phenomenon may be misclassified as progressive disease, the recently modified radiologic, immune-related response criteria (irRECIST, iRECIST) call for a 4-week re-assessment in order to overcome this limitation [42, 43]. In our study, the evaluation of pseudoprogression was partly feasible in the subgroup of 17 patients undergoing a third PET/CT after administration of four cycles of treatment. The comparison between interim and late PET/CT showed signs of pseudoprogression in 2/17 (11.8%) patients, who were characterized as PMD on interim PET/CT according to EORTC and “switched” to MB on late imaging. In contrary, no cases of pseudoprogression were observed after application of PERCIMT, highlighting the ability of the novel criteria in tackling this atypical response pattern. Moreover, the results of survival analysis, exhibiting a lower PFS for patients with early PMD (no-MB) compared to those with early MB, are another indirect proof of the rather low incidence of the phenomenon in this cohort. This is in line with previous results published in the literature documenting non-negligible, but not higher than 10% rates of the phenomenon in melanoma immunotherapy [36, 44, 45], and provides supporting evidence to the standpoint that an increase in tumor burden observed during ICI treatment more likely reflects true progression rather than pseudoprogression [41, 46, 47].

irAEs represent another source of false-positive findings on imaging. Radiologic manifestations of irAEs have been reported with variable incidences, reaching up to 31% of patients under ICIs [47–49]. Although the specific characteristics of individual patients play a significant role, the emergence of these toxicities is mainly dependent on the agents used, with the combination of an anti-CTLA-4 antibody and an anti-PD-1 antibody increasing both their incidence and severity [50]. In the herein studied cohort, 11/25 (44%) of the patients showed signs of irAEs on PET/CT. In line with data from the literature [50], the vast majority of these patients (9/11 patients) received combination treatment of nivolumab and ipilimumab. Colitis was the most frequent adverse event on PET/CT, observed in five patients; four of these patients received PD-1 inhibitors in combination with the anti-CTLA-4 regiment ipilimumab, which is known to often induce this reaction [51, 52]. We recognize that the diagnosis of colitis on PET imaging can be complicated, since its identification can be hampered by physiological metabolic activity in the colon. It is well known that enhanced colon [^18^F]FDG uptake of benign etiology is frequently observed in asymptomatic individuals [53, 54]. Moreover, several studies have shown that patients using the oral hypoglycemic drug metformin tend to have a diffusely increased tracer uptake in the colon [55–58]. In the present cohort, no patient had diabetes; thus, metformin can be ruled out as a cause for false-positive [^18^F]FDG accumulation in the colon. A further search in patients’ clinical history revealed that one of these five patients developed severe diarrhea during immunotherapy, most likely as a symptom of treatment-induced colitis, while the rest four patients did not have such symptoms. This higher incidence of “PET-colitis” under immunotherapy, compared to clinical signs of colitis, is in line with previously published results [59]. In this context, early recognition of radiologic irAEs could be potentially important since they may precede or correlate with clinical symptoms [37, 47], potentially leading to respective changes in management.

Another aspect pertaining to irAEs is that their emergence has been associated with a favorable efficacy of ICIs—mainly of PD-1 inhibitors—implying a potential predictive role of these events for response to ICI treatment [17, 18, 60]. Our analysis revealed that patients with radiologic signs of irAEs had a longer PFS than those without irAEs; however, this difference was non-significant. Apart from the relatively small cohort studied, an explanation for this finding may lie in the fact that most patients (82%) also received the CTLA-4 inhibitor ipilimumab in combination with the PD-1 inhibitor nivolumab; in a recently published meta-analysis of 30 studies including 4971 individuals, it was shown that no significant association between irAE development and a favorable benefit on survival is observed in ICI combination treatments, in contrary to anti-PD-1 monotherapies [60]. Another reason may lie in the nature of the observed irAEs, affecting the gastrointestinal tract in more than half of the cases (6/11 patients, 55%); data from the above-mentioned meta-analysis also highlight the lack of PFS benefit in patients presenting gastrointestinal irAEs [60].

Finally, the predictive role of baseline LDH before initiation of PD-1 inhibitors was investigated. Although serum LDH elevation is not specific for melanoma, it represents a poor prognostic factor and is one of the most influential factors associated with treatment response [61, 62]. An interesting finding of our analysis is the lack of any adverse effect of elevated baseline LDH on survival, which can be however attributed to the small number of patients with pathologic LDH (*n* = 4 patients, 16%).

We note some limitations in our study. Firstly, due to the strict inclusion criteria applied, the number of included patients was relatively low, not allowing us to draw more firm conclusions; ideally, further studies with larger patient cohorts would be required. Secondly, although the focus of the study was anti-PD-1 treatment, not all patients underwent exclusively PD-1 blockade, with several of them receiving combination therapy of PD-1 and CTLA-4 inhibitors. Although our patient cohort is too small to afford a PET/CT subanalysis based on a dichotomization of patients into those receiving anti-PD-1 monotherapy and those undergoing combined treatment, we highlight the similar approach followed in previous studies in the field [28, 63]. Finally, the vast majority of the PET/CT-positive, melanoma-consistent findings were not histopathologically confirmed. However, this is not usually possible in the clinical setting.

## Conclusion

In an attempt to identify early and reliable biomarkers of survival prediction in immunotherapy of metastatic melanoma, we assessed the prognostic role of interim [^18^F]FDG PET/CT performed after the first two cycles of anti-PD-1 treatment. Our results showed that tumor response as classified by the recently proposed PERCIMT criteria is significantly correlated with PFS. This highlights the potential ability of [^18^F]FDG PET/CT for early stratification of response to anti-PD-1 agents, a finding with possible significant clinical and financial implications. Further studies including larger numbers of patients are necessary to validate these results.

## Supplementary information

ESM 1 (DOCX 166 kb)
