# Supplementary material for: Interim [18F]FDG PET/CT can predict response to anti-PD-1 treatment in metastatic melanoma
Source: Eur J Nucl Med Mol Imaging. 2020 Dec 18;48(6):1932–43. doi: 10.1007/s00259-020-05137-7 (PMC8113306; doi:10.1007/s00259-020-05137-7)
Supplement: Supplementary file 1 — (DOCX 166 kb) [file 259_2020_5137_MOESM1_ESM.docx]

**
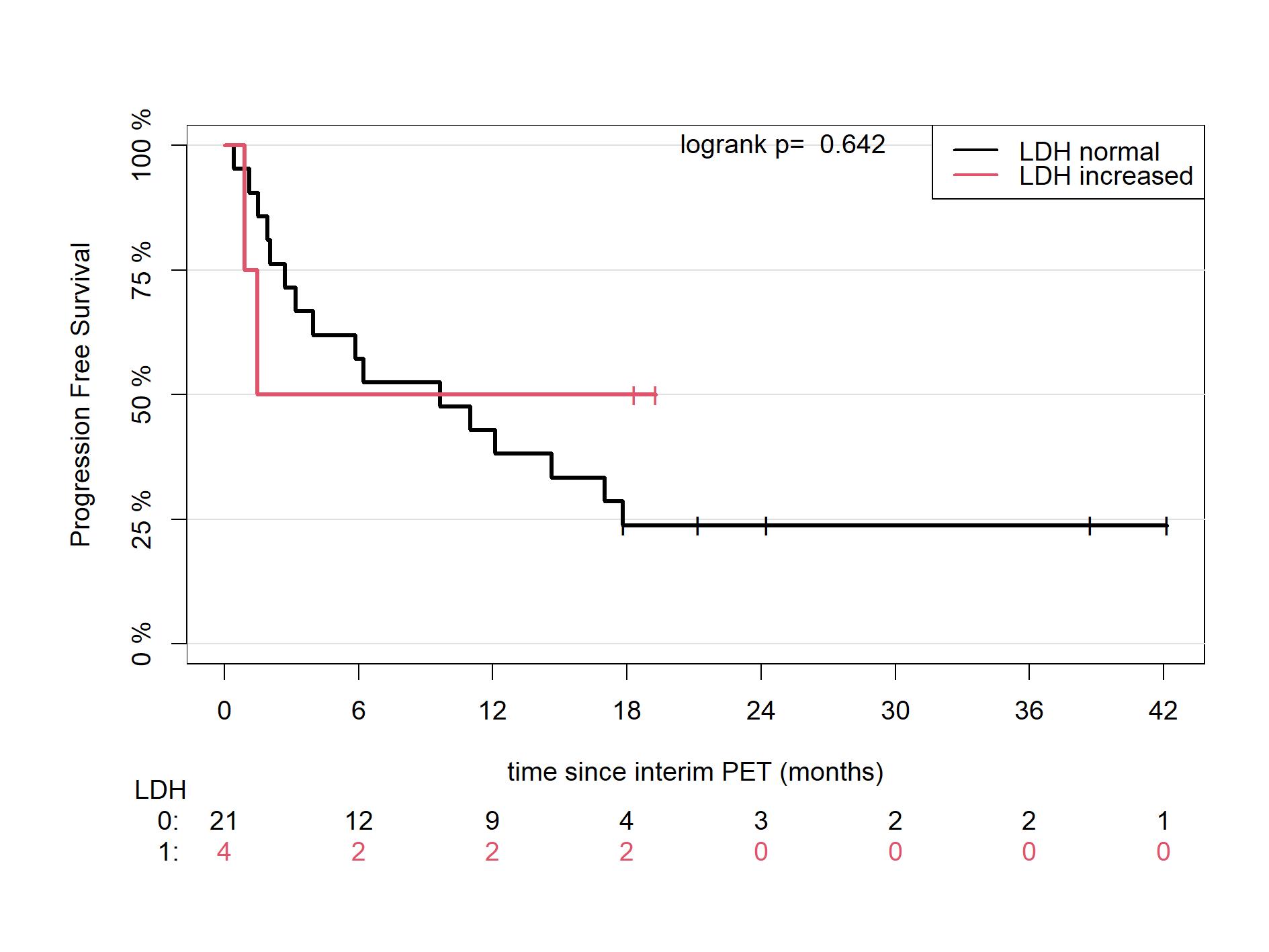
**

**Figure 1** Kaplan-Meier estimates of PFS according to baseline serum LDH. The numbers of patients at risk in each group and for the respective time-points are shown below the plots.
